# How Do Coworkers Aid in Coping with Emotional Exhaustion? An Experience Sampling Method Approach

**DOI:** 10.3390/ijerph16162919

**Published:** 2019-08-14

**Authors:** Jing Xiu, Zhenduo Zhang, Zhigang Li, Junwei Zheng

**Affiliations:** 1 School of Economics, University of Chinese Academy of Social Sciences, Beijing 102488, China; 2 School of Management, Harbin Institute of Technology, Harbin 150001, China; 3 School of Economics and Management, Beijing Polytechnic, Beijing 100176, China; 4 Department of Construction Management, Kunming University of Science and Technology, Kunming 650500, China

**Keywords:** coworker helping behavior, psychological availability, emotional exhaustion, task demands, experience sampling method

## Abstract

The present study emphasizes the indirect influences of coworker helping behavior on emotional exhaustion through psychological availability and the moderating role of perceived task demands on a daily basis. Using a two-wave experience sampling method with data collected via mobile phones, we collected 345 matched data from 69 samples over five consecutive days in mainland China. We developed a moderated mediation model to test our conceptual model, with the following significant results: (1) Daily coworker helping behavior decreased employee emotional exhaustion; (2) daily psychological availability mediated the influence of coworker helping behavior on employee emotional exhaustion; (3) through psychological availability, perceived task demands moderated the indirect influence of daily coworker helping behavior on emotional exhaustion. The indirect influence of daily coworker helping behavior only emerged with a low perception of job demands. This research explores the mechanism and boundary conditions of the relationship between daily coworker helping behavior and employee emotional exhaustion with the job demands-resources model framework. In practice, leaders should adopt beneficial interventions to enhance team cohesion, to facilitate team members’ helping behavior, and to manage task demands.

## 1. Introduction

Employee helping behavior is a provision of the voluntary assistance to coworkers, either to assist with accomplishing goals or to prevent and solve problems [1,2]. Research has mainly focused on the influences of helping behavior on organizations. For instance, Ng and Van Dyne, and Bachrach et al. explored the positive relationship between employee helping behavior and group performance [3,4]. Recent literature has increasingly begun to explore how helping behavior impacts givers’ well-being and work behavior. Koopman, Lanaj, and Scott examined both the positive affective influences and the negative cognitive influences of daily helping behavior toward colleagues on givers’ well-being [5]. Gabriel et al. further explored the depletion process of helping behavior for employees on a daily basis [6]. However, scarce research is performed from the views of employees when they are helped by their coworkers. Considering the transformation of economic system in China, Chinese employees are more likely to face increased emotional job demands and are at high risk to experience emotional exhaustion [7,8]. Thus, the main purpose of the current study is to explain how employee daily well-being (e.g., emotional exhaustion) is affected by coworker helping behavior and provide empirical evidence for the benefits of coworker helping behavior to avoid emotional exhaustion.

To address our research purpose, we developed a multilevel framework by employing an experience sampling method based on the job demands-resources model (JD-R model). JD-R model proposes that there are specific factors correlated with job stress and employee well-being, which are generally divided into two categories—job demands and job resources. Job demands refer to physical, psychological, social, or organizational aspects of the job associated with sustained physical and psychological costs, usually resulting in burnout and decreased well-being at work [9]. In contrast, job resources are those physical, psychological, social, or organizational aspects of the job that help to achieve work goals, reduce job demands, and stimulate personal growth and development [10]. Besides the distinct influences of job demands and job resources, JD-R model addresses the interactive influences of job resources with job demands on employee psychological states and work behavior [9]. For instance, Bakker et al. explored the moderating role of job demands in the positive relationship between job resources and work engagement [11].

**Figure 1:**
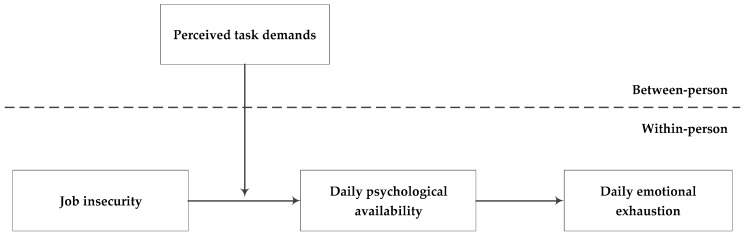
Conceptual model.

Coworker helping behavior, referring to voluntary assistance to recipient colleagues in accomplishing the recipient’s goals or preventing the occurrence of problems, can be regarded as an effective job resource to boost individual’s belief that one has the emotional, physical, or cognitive resources to engage oneself at work (i.e., psychological availability). Further, increased psychological availability will enhance their vigor experienced at work, thereby decreasing emotional exhaustion, denoted as a psychological manifestation of stress that is a facet of job burnout [12]. However, the positive influence of coworker helping behavior is not always effective, which depends on employee perceived task demands according to JD-R model, because of its sustained depletion of personal job resources [11]. This study will explore the indirect influence of daily coworker helping behavior on emotional exhaustion through psychological availability, as well as the moderating role of perceived task demands (See Figure 1).

In doing so, this study will first contribute to the helping behavior literature by examining the influences of coworker helping behavior on employee well-being. This study is developed from the recipients’ perspective, enlarging the scope of previous helping literature. Second, this research contributes to the JD-R model by identifying the mediating role of psychological availability and the moderating role of perceived task demands. This research provides empirical evidence for how, and under which conditions, organizational job resources are transformed into personal job resources, impacting employee emotional exhaustion on a daily basis and offering a more comprehensive insight into the JD-R model.

## 2. Hypothesis Development

### 2.1. Coworker Helping Behavior and Psychological Availability

The aim of coworker helping behavior is to assist the focal recipient to achieve work-related goals or prevent unexpected problems [2]. As a part of social support in the workplace, coworker helping behavior can also be regarded as an effective job resource. The JD-R model addresses the values of job resources for employees, which enable employees to achieve goals and stimulate personal growth and development [13]. Bakker and Demerouti suggested that job resources help people to bolster core self-concept and to fulfil their work-related roles and goals [14]. Decreased job resources impede fulfillment of work roles, leading to greater role stress [15].

Psychological availability is defined as an individual’s belief that they have the emotional, physical, or cognitive resources to engage themselves at work [16]. It assesses the confidence or readiness of employees to engage in their work roles, given the many other roles in which employees must engage [17]. Job resources always exert positive influences when employees are meeting their work requirements. As to the relationship between coworker helping behavior and psychological availability, receiving helping behavior from coworkers simplifies fulfillment of work roles, leading to greater psychological availability for work roles [15]. Further, Binyamin and Carmeli found that uncertainty and stress were critical antecedents to psychological availability [18]. Previous research has revealed an impact of coworker support on reducing uncertainty and work stress [19,20]. Following this logic, we thus proposed the following hypothesis:

**Hypothesis** **1.** *Daily coworker helping behavior increases daily psychological availability.*

### 2.2. Psychological Availability and Emotional Exhaustion

Emotional exhaustion is a state characterized by lack of energy and a feeling of emotional depletion [12]. Previous research suggests that a state of well-being is mainly reflected by the emotional and affective state of a person [21]. Therefore, following the suggestion of van Horn et al. [22], we adopted emotional exhaustion as an indicator of employee wellbeing. In addition, emotional exhaustion has always been regarded as an antecedent to job satisfaction [23], turnover intention [23], and creativity [24].

Role stress (e.g., role ambiguity and role conflict) has been examined as an inducement for emotional exhaustion. Psychological availability is a state in which employees are available for and confident in their work roles [18]. Those with high psychological availability are in a vital psychological condition for engaging in personal work tasks because availability is a sense of security, helping employees to focus on tasks rather than maintain a state of anxiety [16]. Therefore, employees who are psychologically available experience less emotional exhaustion because they are prepared for their work roles, resulting in less role stress, which continuously depletes personal resources and leads to emotional exhaustion [25]. Hence, as consistent with the above arguments, we put forward the following hypothesis:

**Hypothesis** **2.** *Daily psychological availability decreases emotional exhaustion.*

Coworker helping behavior, a beneficial organizational job resource, decreases employee uncertainty and stress, thereby increasing their psychological availability, a helpful personal job resource. Further, increased psychological availability enhances employees’ work role engagement and decreases emotional exhaustion. Previous research has provided empirical evidence for the mediating role of psychological availability in the relationship between organizational resources and positive work behavior [18]. Therefore, we hypothesize the following:

**Hypothesis** **3.** *Daily psychological availability mediates the relationship between coworker helping behavior and emotional exhaustion.*

### 2.3. The Moderating Role of Perceived Task Demands

Previous JD-R research has provided meaningful evidence of the interactive effect of job resources with job demands on employee work-related results (e.g., in-role performance, extra-role performance, well-being) [26,27,28]. Perceived task demands exist when there is a substantial amount of work under great time pressure [29]. In contrast to job resources, job demands exhaust employees’ cognitive and emotional resources and therefore mitigate the positive influences of job resources in the workplace. Thus, this research adopts perceived task demands as a moderator to explore at which boundary condition coworker helping behavior is more or less beneficial for coping with employees’ emotional exhaustion.

Coworker helping behavior provides employees with vital job resources to enhance availability for their work roles [30]. However, when employees experience high task demands, they need to conserve and protect their remaining resources from further depletion, thereby expending fewer job resources into their work roles [31], which buffers the positive influences of coworker helping behavior on psychological availability. In contrast, employee psychological availability is less affected by low perceived task demands, and under such conditions, employees can continue to invest their resources provided by coworker helping behavior to meet the requirements of their work roles.

When employees experience continuous exhaustion of job resources, they will not prioritize their work roles, especially at the expense of other goals, in order to conserve and protect their job resources [31]. Ng, Ang, and Chan have provided empirical evidence for the moderating effects of task demands on the relationship between leaders’ positive personalities and self-efficacy [32]. Thus, we propose the following hypothesis:

**Hypothesis** **4.** *Perceived task demands moderate the relationship between daily coworker helping behavior and psychological availability.*

Employees with high task demands will choose to conserve their job resources, leading to the investment of fewer resources obtained from coworker helping behavior to their work roles, thereby decreasing psychological availability and increasing emotional exhaustion. In contrast, the indirect influence of coworker helping behavior on emotional exhaustion through psychological availability will be less affected by low task demands. Thus, we propose the fifth hypothesis:

**Hypothesis** **5.** *Perceived task demands moderate the indirect effect of daily coworker helping behavior on emotional exhaustion through psychological availability.*

## 3. Methods

### 3.1. Sample and Procedure

**Figure 2:**
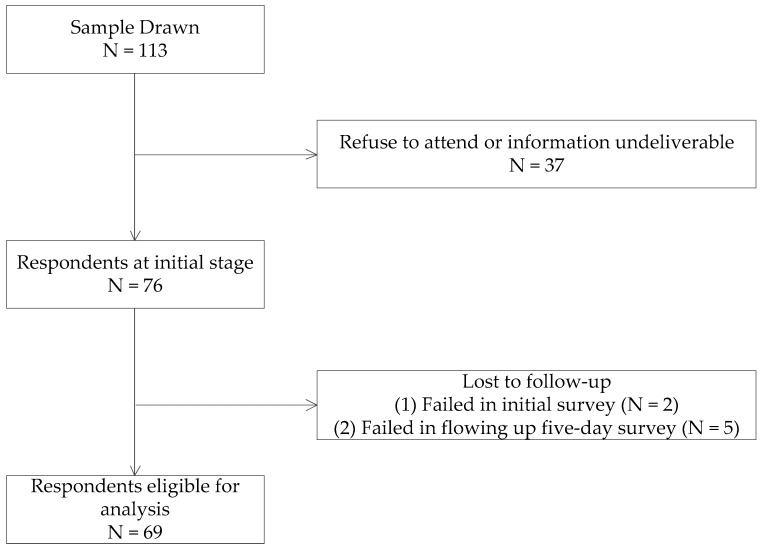
Flowchart of samples identified and included in studies.

To explore the dynamic influences of daily coworker helping behavior on emotional exhaustion, we adopted an experience sampling method to collect daily data. We developed a subject pool with 113 alumni of our college in a university in Beijing, China, who had updated their contact information within the past two years with help from our college secretary. All participating alumni satisfied two requirements: worked full-time hours (above 40 h) per week and worked in mainland China. We contacted the initial samples through e-mail, telephone, and WeChat, a mobile social app, to enquire whether or not they were willing to participate in the survey. We explained the research purpose and five-day research process. Finally, 76 of 113 samples confirmed their participation and we formed a research group on WeChat. On the first Sunday, they received a web link of the initial survey, including code, gender, education, age, and perceived task demands. On the following five work days, from Monday through Friday, we sent them a questionnaire on WeChat at 11:00 a.m. (assessing coworker helping behavior and psychological availability) and at 6:00 p.m. (assessing emotional exhaustion). Participants were given one hour to complete each questionnaire via a mobile website. In the end, a total of 345 valid questionnaires from 69 participants were returned for analysis, of which two samples didn’t finish the initial survey and five samples failed to follow-up on the five-day survey. The effective response rate was 90.79%. The flowchart of samples was shown in Figure 2.

### 3.2. Approval of the Research Protocol

The study procedures were approved by the Ethics Committee of Kunming University of Science and Technology (skpyyb201721) and were in line with the 1964 Helsinki Declaration and its later amendments or comparable ethical standards. Informed consent was signed and obtained from all individual participants included in the study.

### 3.3. Measures

All study items were adopted from top peer-reviewed journals originally in English and were translated into Chinese following a formal back-translation procedure [33]. Response of this study were obtained on a five-point Likert scale, with 1 indicating strongly disagree and 5 indicating strongly agree.

Coworker helping behavior was measured by a three-item scale developed by Yue, Wang, and Groth [2]. A sample item was “Today, my coworkers helped me when my work-load was high.” Participants were required to finish this questionnaire based on their morning work experience. The scale yielded a Cronbach’s alpha = 0.84.

Psychological availability was measured by three items developed by Byrne, Peters, and Weston [34]. A sample item was “Today, I am emotionally ready to deal with the demands of my work.” The internal consistency of this scale was 0.91.

Emotional exhaustion was measured by three items developed by Watkins et al. [12]. A sample item was “Today, I feel emotionally drained from my work.” The Cronbach’s alpha of this scale was 0.87.

Perception of task demands was measured by three items developed by Williams and Alliger [29]. A sample item was “I am rushed or hurried to complete my recent activity”. They were required to finish this questionnaire according to their most recent month of work experience. This scale yielded a Cronbach’s alpha = 0.88.

Control variables. Based on previous research, we included gender (0 = male, 1 = female), education (1 = college or below, 2 = bachelor degree, 3 = master’s degree or above), and age (years), because of their potential influences on emotional exhaustion [35,36].

## 4. Results

### 4.1. Multilevel Confirmatory Analysis and Descriptive Statistics

A multilevel confirmatory factor analysis (MCFA) was applied to test the validity of the four-factor construct. At the within-person level, we included learning, emotional exhaustion, and psychological availability. At the between-person level, we included job insecurity. The hypothesized four-factor conceptual model showed a better fit for the data (χ^2^(24) = 45.30, RMSEA = 0.05, RMR = 0.03, CFI = 0.99) than did the baseline two-factor construct (△χ^2^(3) = 565.01, *p* < 0.01). Table 1 further provides the descriptive statistics, intro-correlations, and correlations of the study variables.

Table 2 showed the results of descriptive statistic and correlation analysis. Participants were mainly in financial services, electronic, and manufacturing industries: 46.4% of the participants were males; 75.4% of the participants held bachelor’s degrees and college certifications; the mean age of participants was 29.05 ± 4.70.

At the within-person level, coworker helping behavior increased psychological availability (*r* = 0.39, *p* < 0.01) and decreased emotional exhaustion (*r* = −0.39, *p* < 0.01). Psychological availability was associated with decreased emotional exhaustion (*r* = −0.63, *p* < 0.01).

### 4.2. Regression Analysis

The first stage of statistical analysis investigated systematic within- and between-person variance for the daily variables. The episodic variance for emotional exhaustion, psychological availability, and coworker helping behavior were 0.35, 0.41, and 0.31 respectively, which judged the adoption of hierarchical regression analysis.

Hypotheses were examined via a multilevel moderated mediation analysis, using the HLM software (Version 6.08, Scientific Software International, Inc., Skokie, IL, USA). Before we ran hierarchical linear regression equations, within-person variables were all group centered, while some of the between-person variables (age and perceived task demands) were grand centered.

Table 3 shows the results of Model 1 and Model 3 indicating that coworker helping behavior was associated with increased daily psychological availability (*γ* = 0.20, *p* < 0.01) and decreased emotional exhaustion (*γ* = −0.17, *p* < 0.05), supporting Hypothesis 1. When daily emotional exhaustion was regressed on daily psychological availability and coworker helping behavior simultaneously, only daily psychological availability (*γ* = −0.16, *p* < 0.01) was significant, whereas coworker helping behavior (*γ* = −0.12, n.s.) was not significantly associated with daily emotional exhaustion in Model 4. Further, through the Monte Carlo bootstrapping test using Rstudio (Version 3.5.3, RStudio, Inc., Boston, MA, USA), the indirect effect of job insecurity on daily emotional exhaustion through daily psychological availability was significant (indirect effect = −0.03, 95% confidence interval = [−0.08, −0.01]), supporting Hypotheses 2 and 3.

**Figure 3:**
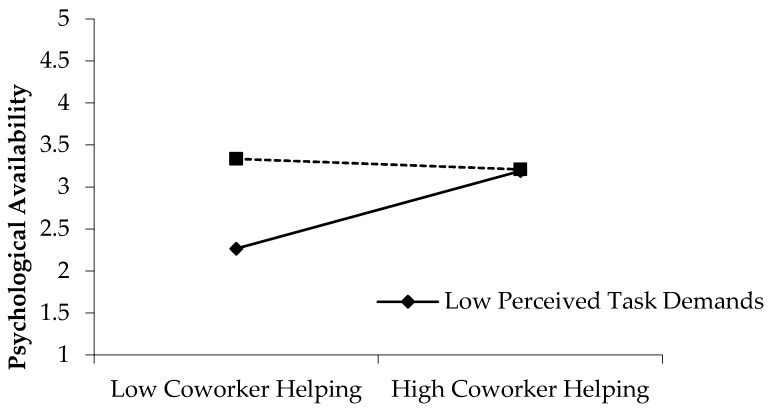
The moderating role of perceived task demands.

The Model 2 results showed that the interactive item of daily coworker helping behavior with perceived task demands was associated with decreased daily psychological availability (γ = −0.26, *p* < 0.05). To further directly elaborate the moderating role of daily learning, Figure 3 was developed based on a method proposed by Aiken and West (1999), indicating that when perceived task demands were low, the positive effect of daily coworker helping behavior on psychological availability was significant (effect = 0.40, 95% confidence intervals = [0.18, 0.62]). Conversely, when daily learning was high, the positive relationship between daily coworker helping behavior and psychological availability was mitigated (effect = 0.00, 95% confidence intervals = [−0.22, 0.22]). Hypothesis 4 was supported.

Moreover, we calculated their Monte Carlo confidence intervals at different combinations of higher and lower values of the moderators using Rstudio (Version 3.5.3, RStudio, Inc., Boston, USA). In Table 4, daily coworker helping behavior had a negative indirect effect on the daily emotional exhaustion through daily psychological availability only when perceived task demands were low (indirect effect = −0.06, 95% confidence intervals = [−0.13, −0.01]), whereas the indirect effect disappeared when perceived task demands were high (indirect effect = 0.00, 95% confidence intervals = [−0.04, 0.04]). Hypothesis 5 was supported.

## 5. Discussion

This research examines the indirect influences of coworker helping behavior on employee emotional exhaustion through psychological availability at the episode level. Further, the indirect influence is significant only when perceived task demands are low. When perceived task demands increase, the positive indirect influence of coworker helping behavior disappears.

This finding is consistent with previous research, which addresses the helpful influences of job resources on meeting employee goals while decreasing associated psychological costs [13]. This research identifies coworker helping behavior as a beneficial organizational job resource that can be transformed into personal job resources, thereby decreasing emotional exhaustion. Further, our research explores the boundary conditions under which organizational job resources can be transformed into personal resources by examining the moderating role of perceived task demands, which provides daily empirical evidence for the JD-R model.

### 5.1. Theoretical and Practical Implications

Our research has two theoretical implications for both helping literature and the JD-R model. First, by incorporating coworker helping behavior, our research extends the scope of helping behavior literature. Previously, helping behavior literature mainly focused on two domains. The first stream is to explore how employee helping behavior fosters organizational effectiveness. For instance, Bachrach et al. addressed the relationship between helping behavior and group performance [4]. The other stream is to elaborate the psychological and behavioral outcomes of focal helpers. By adopting experience sampling method, Gabriel et al. revealed the depleting process of helping behavior, that was daily helping behavior would cause depletion for helpers and led to less helping behavior in the next day [6]. However, rare research was performed from the perspectives of helping recipients.

Employees can count on their colleagues to help and support them when they are experiencing high workloads [37]. Coworker helping behavior may increase employees’ sense of emotional work satisfaction, which, over time, decreases the emotional exhaustion [38]. In this vein, this research examined the positive emotional impacts of coworker helping behavior on employees, the results of which were consistent with previous research, which recognized coworker helping behavior as an organizational job resource. Regardless of the previous organizational citizenship behavior (OCB), helping behavior literature studies the influences of helping behavior on organizational effectiveness [3], and on the givers of helping behavior [5]. Our research extends this to offer a new perspective for helping behavior research.

Second, our research unveils the mechanism and boundary condition for the transformation process from organizational job resources to personal job resources at the episode level, which provides a more comprehensive insight into the JD-R model. JD-R model addressed the positive influences of job resources for employees. They reduced the psychological costs associated with task demand, were functional in helping employees achieved work goals, and motivated employees to seek for development and growth in organizations [13,26,39]. Research has divided job resources into different categories, such as coworker helping behavior at the organizational level and psychological availability at the individual level [40,41]. However, little research has been done to examine the influences of different categories of job resources on each other. For example, Hu, Schaufeli, and Taris [42] examined distinct influences of social resources, guanxi exchange, and task resources on burnout engagement and organizational commitment in the Chinese context. This research examined the organizational job resources (e.g., coworker helping behavior) that were transformed into personal job resources (e.g., psychological availability) and used to enhance employees’ positive emotional experiences, thereby decreasing their emotional exhaustion on a daily basis.

Further, our research reveals that the transformation process can be realized only under the condition of low perceived job demands because high job demands will continuously deplete resources obtained from coworkers, which leaves fewer resources to be transferred into psychological availability. Both the direct and interactive effects of job demands and job resources on various psychological outcomes have been paid lots of attention [43]. Indeed, JD-R model proposes that job resources boots work-related well-being, whereas task demands are associated with decreased well-being [10,14]. The job stress literature suggests the reliable relationship between mental health problems and task demands. Task demands continually deplete job resources, and then exert influences on sequential job resources and work-related outcomes [44]. Zhang et al. uncovered the buffering influence of task demands on the positive relationship between job resources (i.e., job autonomy) and creativity [45]. Besides uncovering the transformation process from organizational job resources (i.e., coworker helping behavior) to personal job resources (i.e., psychological availability), our research further explores the boundary condition of this process by examining the buffering role of perceived task demands, which contributes new knowledge to the JD-R model.

Our research also has practical implications. First, negative perceptions of work conditions and colleague relationships hinder employees’ willingness to help each other. Previous research has found that leaders play a vital role in promoting and nurturing team cohesion. Leaders should strive to nurture an agreeable team climate in which employees are willing to help each other [46]. For instance, Senecal, Loughead, and Bloom provided a plan of team-building interventions through goal setting to enhance team cohesion [47]. Second, our research addresses the negative impacts of perceived task demands. To better optimize the influences of coworker helping behavior, organizations should promote job crafting, a means by which employees can change the basic elements and social environments of their jobs, resulting in increased job resources and decreased job demands [14]. Wingerden, Bakker, and Derks proposed a job demands-resources intervention, whose core component is job crafting, in order to manage employee personal task demands [48]. For the metro policy, the Natural Science Foundation of China called for research facilitating the establishment of harmony and innovative organizational climate. Indeed, group harmony plays the central role in enhancing team cohesion and effectiveness [49,50]. Further, Larson et al. addressed that organizational climate can be shaped through intervention [51]. Thus, organizations should pay attention to and adopt effective intervention to establish harmony in the organizational climate.

### 5.2. Limitations and Future Research

Our research has some limitations that suggest directions for future research. First, we cannot establish firm causal relationships between our focal variables. Although we collected two-wave data (in the morning and in the afternoon) over five consecutive days, we still cannot rule out a reversed causal relationship, especially for the relationship between emotional exhaustion and psychological availability. Experimental and cross-lagged design have been regarded as the effective tools that infer causal effect [52,53]. For instance, Gils et al. used scenario-based experiment to manipulate ethical leadership and moral attentiveness to examine the interactive effect on follower organizational deviance [54]. Besides, Viotti et al. adopted a one-year cross-lagged design to test the reciprocal associations among co-worker incivility, organizational inefficiency, and work-related exhaustion [55]. For future research, a cross-lagged panel design or an experimental design may be considered in order to further explore the causal effect of our conceptual model [52,53].

Second, we cannot avoid common method variance (CMV; Podsakoff, Mackenzie, Leem, and Podsakoff [56]) completely. Our two-wave experience sampling method design can decrease the influences of CMV to a certain degree; however, considering our self-reported questionnaires, there are still potential biases caused by CMV. Podsakoff et al. [56] has suggested some effective ways to decrease the bias of CMV, one of which is to adopt multi-source data, which is widespread in the research field of organizational behavior [57,58]. For future research, multi-source data may be adopted to rule out CMV. For instance, perceived task demands may be indicated by objective job demands.

Third, there are paradoxical insights into the influences of perceived task demands. For instance, Williams and Alliger regarded the perception of task demands as a component of work stress, which correlated positively with distress [29]. In contrast, Schyns and Croon studied the increased task satisfaction [59]. Thus, the double-edged characteristic of perceived task demands can be inferred. Although our research is consistent with previous research in uncovering the buffering impact of perceived task demands on the positive relationship between coworker helping behavior and psychological availability, the negative effect of perceived task demands on emotional exhaustion was significant (*γ* = −0.44, *p* < 0.05), revealing the positive side of perceived task demands. To uncover the characteristic of perceived task demands, future research can examine whether perceived task demand is a challenge or a hindrance stressor within the framework of conservation of resources theory. If perceived task demand is a challenge stressor, it can facilitate employee work engagement and task performance [60,61]. However, if it is a hindrance stressor, it will result in high workload and job strains [62]. Future research should investigate the complex insight into the influences of perceived task demands, which will offer us more beneficial strategies to optimize job demands and facilitate employees’ growth within organizations.

Fourth, our research sample size was relatively small. This research adopted 345 matched data from 69 persons over five consecutive days. Although there were no specified criteria for the sample size in the experience sampling method design, it will be better for future research to collect daily data from more samples. It is difficult for small samples to perform heterogeneity analysis across groups (e.g., education and age.), which will not rule out the potential bias caused by the selection of our samples, because the employees are not randomly assigned into workplaces. Böckerman et al. suggested that using information on employees’ wage and work histories may be a solution to the bias [63]. We have controlled education and age, which were associated with employee’s wage and work histories [64,65], however, it will be better for future research to control the two variables directly in their research. Further, some other psychological variables (e.g., personality), which indicated individual differences, should be also adopted in the regression model to decrease the omitted-variable bias and improve the reliability of the results.

## Figures and Tables

**Table 1:** Results of confirmatory factor analysis.

Models	Variables	χ^2^	*df*	△χ^2^	RMSEA	RMR	CFI
Four-Factor	CH, PSYA, EE, PT	45.30	24		0.05	0.03	0.99
Alternative Model							
Three-Factor	CH+PSYA, EE, PT	393.33	26	348.03 **	0.20	0.13	0.82
Three-Factor	CH+EE, PSYA, PT	359.21	26	313.91 **	0.19	0.11	0.84
Three-Factor	CH, EE+PSYA, PT	291.12	26	245.82 **	0.17	0.08	0.87

Note: CH = coworker helping behavior, PSYA = psychological availability, EE = emotional exhaustion, PT = perceived task demands; ** *p* < 0.01.

**Table 2:** Descriptive statistics and correlation analysis.

**Between-Person (*n* = 69)**	**Mean**	**SD**	**1**	**2**	**3**	**4**
1. Gender	-	-	-			
2. Education	-	-	0.08	-		
3. Age	29.06	4.72	0.00	−0.23 *	-	
4. Perceived Task Demands	3.05	0.78	0.05	0.06	0.05	(0.88)
**Within-Person (*n* = 345)**			**1**	**2**	**3**	
1. Coworker Helping Behavior	3.18	0.96	(0.84)			
2. Psychological Availability	3.82	0.78	0.39 **	(0.91)		
3. Emotional Exhaustion	2.64	0.91	−0.39 **	−0.63 **	(0.87)	

Note: values in the parenthesis are Cronbach’s alpha. * *p* < 0.05; ** *p* < 0.01.

**Table 3:** Results of hierarchical linear model analysis.

Variable	Psychological Availability				Emotional Exhaustion			
	Model 1		Model 2		Model 3		Model 4	
	γ	SE	γ	SE	γ	SE	γ	SE
Intercept	3.90	0.35	3.95	0.37	2.90	0.53	2.71	0.43
Between-Person (*n* = 69)								
Gender	−0.15	0.14	−0.17	0.13	0.17	0.17	0.23	0.17
Education	0.07	0.13	0.06	0.15	−0.25	0.20	−0.20	0.16
Age	−0.02	0.01	−0.02	0.01	0.02	0.02	0.02	0.01
PT			0.27	0.17			−0.40 **	0.11
Within-Person (*n* = 345)								
CH	0.20 **	0.07	0.20 **	0.07	−0.17 *	0.08	−0.12	0.08
PSYA							−0.16 **	0.06
Interaction								
CH × PSYA			−0.26 *	0.12			−0.01	0.12
Pseudo R^2^	0.09		0.14		0.03		0.17	
−2LL	631.29		626.34		705.81		693.01	

Note: CH = coworker helping behavior, PSYA = psychological availability, EE = emotional exhaustion, PT = perceived task demands; −2LL = −2LogLikelihood * *p* < 0.05; ** *p* < 0.01.

**Table 4:** Results of the Monte Carlo bootstrapping test.

**Moderating Effect**	**Effect**	**SE**	**95%LLCI**	**95%ULCI**
Low Perceived Task Demands	0.40	0.11	0.18	0.62
High Perceived Task Demands	0.00	0.11	−0.22	0.22
**Moderated Mediation Model**	**Indirect Effect**	**SE**	**95%LLCI**	**95%ULCI**
Low Perceived Task Demands	−0.06	0.03	−0.13	−0.01
High Perceived Task Demands	0.00	0.01	−0.04	0.04
Difference	0.06	0.04	0.01	0.15
**Path**	**Effect**	**SE**	**95%LLCI**	**95%ULCI**
Coworker Helping Behavior	−0.13	0.10	−0.31	0.05
Coworker Helping Behavior →Psychological Availability →Emotional Exhaustion	−0.03	0.02	−0.08	−0.01
